# Editorial: Cognitive and Personality Variables in the Development of Behavioral Addictions in Adolescence and Emerging Adulthood

**DOI:** 10.3389/fpsyg.2022.892669

**Published:** 2022-04-08

**Authors:** Alessia Passanisi, Ugo Pace, Luca Milani, Adriano Schimmenti

**Affiliations:** ^1^Department of Human and Social Sciences, Kore University of Enna, Enna, Italy; ^2^Centre for Research Into Developmental and Educational Dynamics, Department of Psychology, Catholic University of Milan, Milan, Italy

**Keywords:** behavioral addiction, cognitive variable, personality, adolescence, emerging adult

As part of the scientific debate on the nature of behavioral addictions, it has been shown the tendency of researchers to elaborate, for the lack of theoretical and clinical conceptualizations, evidence related to disorders by adapting the criteria for addictive disorders linked to substance abuse, assuming that there is a sort of conceptual overlap or similarity. Indeed, some researchers have observed that people characterized by behavioral addictions show symptoms related behaviors similar to those of people with substance use disorders, such as compulsive behaviors, frequent and obsessive thoughts about a particular activity, to the exclusion of other social interests', psychological distress when the activity is reduced, reduction in social, recreational, professional, educational, and domestic activities.

Especially in the period from puberty to early adulthood, some behaviors generally considered adaptive as gaming, physical activity or buying can assume a particular meaning in the psychosocial development of the individual (Milani et al., 2018; Triberti et al., 2018; Ruggieri et al., 2020). Moreover, behaviors that are moderately maladaptive, such as gambling, may exacerbate and compel adolescents into a clear addiction. The risk, in this case, is to generate a condition in which psychological dependence pushes the craving for the object, without which, the very existence loses its primary meaning. Thus, the displacement of psychic energies on the object of the addiction itself and the complementary withdrawal from life without the object of addiction, experienced as monotonous, can become real risk factors in lives of adolescents and young adults (Pace et al., 2019). For individuals in this stage of development, these behaviors could provide ideal ground for virtual transposition of points of view, dreams, desires, but also hardships and existential problems.

This Research Topic aims to deepen the knowledge about the conditions under which compulsiveness, craving, pleasure gained or loss of control act as a catalyst for compensating those psychosocial needs that are not met in adolescence and emerging adulthood, leading to so-called “new addiction.” Due to their condition of fragility linked to the particular developmental period, adolescents and young adults represent the population most at risk of behavioral or social addictions.

Recently, in the psychology literature, a great amount of empirical research on behavioral addictions among adolescents and young adults has led to the suggestion that there might be a general predisposition toward addiction in some individuals characterized by neuro-biological abnormalities which inhibit the “rational brake” in specific cortical areas that regulate the ability to make rational decisions. Others researchers have tried to go beyond the simple concept of predisposition, suggesting that the complex factors linked to a behavioral addictions would result from scarcity in a multiplicity of cognitive and emotional devices associated with decision making. Beyond studies on neural circuitry, considered central to neurobiological models of pathological behaviors, remains the dilemma of how to understand the relationship between predisposing characteristics of personality and processes of decision making in individuals with addiction.

Extensive research has shown that behavioral addictions and personality disorders are highly comorbid in both treatment and community research studies. The DSM-5 defines a personality disorder as “an enduring pattern of internal experience and markedly deviating behavior of the expectations of the culture of the individual, it is pervasive and inflexible.” The impact of maladaptive personality traits on play is an expanding Research Topic. Several authors have explored the role of personality dimensions in the development of gambling disorder using the Big Five Model e the five-factor model (FFM). In particular, several studies have shown greater neuroticism and lower scores of agreeableness, conscientiousness and openness among behavioral addicted people. This suggests that people with problem behavior are more likely to be less imaginative, intellectually curious, orderly, responsible, and open-minded.

Moreover, according to psychological literature, the process of deciding on the basis of instantaneous reward or, conversely, on postponed but elevated reward, is considered a decisive point on an adaptive psychosocial developmental trajectory. An adaptive decision-making function should be based on the postponement of impulsive urges for immediate gratification and persistence in goal-directed behavior to achieve positive outcomes in the future. This model outlines a complex set of cognitive mechanisms that encourage persistent maladaptive behaviors (Passanisi et al., 2017). The power and occurrence of irrational cognitive beliefs get stronger with increasing levels of a behavioral addiction: poor critical thinking would be involved in the development and maintenance of the different behaviors that, although socially accepted, become the fulcrum of the adolescent's life due to their pervasiveness and excessive interest at the expense of any other adolescent development task.

The research presented in this topic highlights some interesting results for the understanding of behavioral addictions among adolescents and young adults. In particular, some subjects of these studies were: gaming and gambling, seen in their opposite meanings of moment of leisure or addiction and through their personality correlates; physical activities, as protective factors, in the case of addiction related to substances, or as risk factors; and, finally, buying and cosmetic treatments, with respect to its increasingly compulsive meaning in our society.

Taken as a whole, results highlight the pervasiveness of the developmental risks connected with the loss of control on those behaviors that can be thought as non-atypical *per se*, but also the array of potential protective factors that can buffer the maladaptive trajectories of adaptation into healthier outcomes. Nonetheless, the need for a comprehensive theoretical and clinical model about “new addictions” emerges clearly. This is likely to be the challenge that lays ahead for the upcoming research.

## Author Contributions

All authors listed have made a substantial, direct, and intellectual contribution to the work and approved it for publication.

## Conflict of Interest

The authors declare that the research was conducted in the absence of any commercial or financial relationships that could be construed as a potential conflict of interest.

## Publisher's Note

All claims expressed in this article are solely those of the authors and do not necessarily represent those of their affiliated organizations, or those of the publisher, the editors and the reviewers. Any product that may be evaluated in this article, or claim that may be made by its manufacturer, is not guaranteed or endorsed by the publisher.
